# “Picolog,” a Synthetically-Available Bryostatin Analog, Inhibits Growth of MYC-Induced Lymphoma *In Vivo*

**DOI:** 10.18632/oncotarget.438

**Published:** 2012-02-02

**Authors:** Brian A. DeChristopher, Alice C. Fan, Dean W. Felsher, Paul A. Wender

**Affiliations:** ^1^ Departments of Chemistry and Chemical and Systems Biology, Stanford University, Stanford, CA 94305-5080; ^2^ Division of Oncology, Stanford University School of Medicine, Stanford, CA 94305

**Keywords:** bryostatin, picolog, lymphoma, PKC

## Abstract

Bryostatin 1 is a naturally occurring complex macrolide with potent anti-neoplastic activity. However, its extremely low natural occurrence has impeded clinical advancement. We developed a strategy directed at the design of simplified and synthetically more accessible bryostatin analogs. Our lead analog, “picolog”, can be step-economically produced. Picolog, compared to bryostatin, exhibited superior growth inhibition of MYC-induced lymphoma *in vitro*. A key mechanism of picolog's (and bryostatin's) activity is activation of PKC. A novel nano-immunoassay (NIA) revealed that picolog treatment increased phospho-MEK2 in the PKC pathway. Moreover, the inhibition of PKC abrogated picolog's activity. Finally, picolog was highly potent at 100 micrograms/kg and well tolerated at doses ranging from 100 micrograms/kg to 1 milligram/kg *in vivo* for the treatment of our aggressive model of MYC-induced lymphoma. We provide the first *in vivo* validation that the bryostatin analog, picolog, is a potential therapeutic agent for the treatment of cancer and other diseases.

## INTRODUCTION

**Figure 1:**
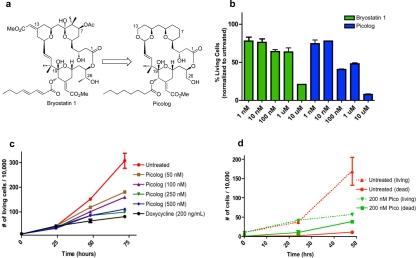
(a) Structures of bryostatin 1 and the structurally simplified (although comparably or more potent) analog “picolog.” (b) Picolog vs. bryostatin 1 dose response. Percentage of viable 4188 lymphoma cells is graphed (normalized to untreated controls) following 36 hour incubation with 1 nM, 10 nM, 100 nM, 1 μM, or 10 μM bryostatin 1 (green) or picolog (blue). Each bar represents the average of two values normalized to the average of two untreated control cell populations. P values (two-tailed unpaired t test): 0.664 (1 nM), 0.750 (10 nM), 0.010 (100 nM), 0.096 (1 μM), 0.001 (10 μM). (c) Cell growth over time for untreated 4188 cells as well as those treated with picolog (50 nM, 100 nM, 250 nM, 500 nM) and doxycycline (200 ng/mL). Each point represents the average of two values. P values (72 hour time points, two-tailed unpaired t test compared to untreated): 0.054 (50 nM), 0.040 (100 nM), 0.022 (250 nM), 0.023 (500 nM), 0.018 (dox). (d) Cell growth (dotted lines) and death (solid lines) over time for untreated (red) 4188 cells as well as those treated with 200 nM picolog (green). Dead cells are defined as those staining positive for trypan blue, while living cells are defined as those excluding trypan blue. Each point represents the average of two values. P values (48 hour time points, two-tailed unpaired t test): 0.091 (living), 0.001 (dead).

Bryostatin 1 (Figure 1a), isolated from an extract of the marine bryozoan *Bugula neritina*, is the prototypical member of a family of complex macrolides that has garnered significant attention from chemists, biologists, and clinicians owing to its structural novelty and remarkable biological activities [1-4]. These activities include induction of apoptosis [2], reversal of multidrug resistance [2], immunogenic stimulation [5], enhancement of memory and cognition in animal models [6], postischemic neuronal rescue and synaptogenesis [7-8], and *in vitro* activation of latent HIV reservoirs [9-10]. Thus far, bryostatin has been used in Phase I and II clinical trials against several types of cancers, both as a single agent and, more recently, in combination with other cancer chemotherapeutics [11-13]. It is also being evaluated in a recently opened trial for Alzheimer's treatment (see http://clinicaltrials.gov). Remarkably, bryostatin 1 is so potent that only ~1 mg is needed for a 16-week course of treatment of patients in cancer clinical trials [14]. The activity profile of bryostatin 1 makes it an excellent candidate for the treatment of a number of diseases that are considered the most significant global health challenges, including neurodegenerative disorders, HIV/AIDS, and cancer.

Despite the enormous potential of bryostatin 1 as a therapeutic agent, its clinical advancement and the search for even more effective derivatives have been hampered by its natural scarcity; a large scale GMP isolation, for example, provided only 18 grams of bryostatin from 14 tons of *B. neritina* [1]. Moreover, the natural product is difficult to modify as is needed to tune its selectivity toward targeting distinct molecular pathways and minimizing off-target toxicities. While significant progress has been made on alternative sources, aquaculture, engineered biosynthesis [15], and total synthesis [16-24] have not yet proven practical as a supply source. As a result, most preclinical and clinical work has been conducted with the dwindling supply of bryostatin 1 obtained in the original GMP isolation. A further complication arises from the low *in vivo* dosages of bryostatin and thus low plasma levels, which often prohibit traditional pathway and pharmacokinetic analyses owing to instrumental limits of detection [25]. The limited supply of bryostatin 1 and the absence of comparably potent and potentially more effective agents have slowed exploitation of this clinically promising lead. Given that bryostatin is neither evolved nor optimized for human therapeutic use, the design and synthesis of simplified and thus more synthetically accessible analogs that could exhibit superior clinical performance are goals of considerable immediate significance. Indeed, patient accrual in a recent clinical trial involving bryostatin was terminated early due, in part, to the potential of “more potent bryostatin analogs in development” [13].

Starting in the 1980s, the Wender group synthesized a number of bryostatin analogs that were designed for ease of synthesis and superior clinical performance [3, 26]. Significantly, several of these analogs display comparable or even superior activity when compared to bryostatin 1 in *in vitro* models for both cancer and Alzheimer's disease [4, 26-29]. One such analog, termed “picolog” (Figure 1a), is a lead analog in the Wender library across all *in vitro* data sets and can be prepared in only 29 synthetic steps in quantities sufficient to supply preclinical studies and clinical trials [27]. Given the promising *in vitro* performance of this analog, evaluation of its *in vivo* performance is now a critical step for preclinical advancement. This current study marks the first *in vivo* investigation of this promising analog.

We had several considerations in selecting a preclinical model for this initial *in vivo* administration of picolog. The natural product bryostatin 1, a modulator of protein kinase C (PKC) activity, has been shown to affect MYC regulation in leukemias and neuroblastomas [30-33]; therefore, we chose to study the activity of picolog in a MYC-induced neoplasm.

The Felsher laboratory has generated numerous conditional transgenic models of hematopoietic and epithelial malignancies that overexpress human c-MYC in specific tissue compartments [34-41]. Transgenic models have been invaluable for determining the role of MYC in tumor maintenance as well as for investigating potential efficacy of novel therapeutics against MYC-induced cancer [34-42]. Furthermore, although bryostatin 1 efficacy has been demonstrated *in vitro* and *in vivo* against a number of different neoplasms as both a single agent and as part of a combination regimen, 28% of all clinical trials involving bryostatin 1 have been for the treatment of lymphoma (see http://clinicaltrials.gov) [43-44]. Thus, to study the potential efficacy of picolog in an *in vivo* model with direct clinical implications, we selected the conditional transgenic model of a rapidly progressive lymphoma arising from MYC overexpression in the lymphoid compartment [34-41]. Using this model, we demonstrate for the first time that picolog administered *in vivo* is well tolerated and can inhibit the growth of aggressive, MYC-induced lymphoma.

## RESULTS

### Picolog is more potent than bryostatin 1 *in vitro*

The activity of picolog was compared to bryostatin 1 *in vitro*. Concentrations ranging from 1 nM-10 μM were administered to a murine tumor-derived lymphoma cell line over 36 hours (Figure 1b). At all concentrations tested, picolog exhibited comparable or superior growth inhibition of lymphoma cells relative to bryostatin 1 (p < 0.05 for 100 nM and 10 μM treatment groups).

**Figure 2:**
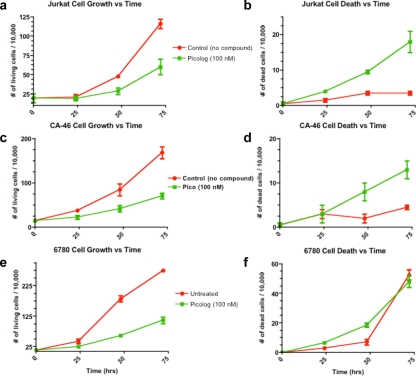
Cell growth and cell death over time for a panel of additional leukemia and lymphoma cell lines (Jurkat (T-cell leukemia), CA-46 (Burkitt's lymphoma), and 6780 (transgenic MYC-induced lymphoma)) treated with 100 nM picolog.

Over a 72 hour time period, cell growth curves of lymphoma cells treated with 50-500 nM picolog revealed a dose-dependent growth inhibition (Figure 1c). At the highest concentrations tested, (250 nM, 500 nM), picolog treatment was as effective at inhibiting cell growth as MYC inactivation with doxycycline. In addition to growth inhibition, a statistically significant (p < 0.05) increase in cell death was observed following treatment with 200 nM picolog for 48 hours (Figure 1d). To determine if picolog inhibited growth of additional murine and human MYC-induced lymphoma cells, 100 nM picolog was administered to a panel of additional T- and B-cell lymphomas of human and murine origin (including human Jurkat T-cell lymphoma, CA-46 B-cell lymphoma, and 6780 murine T-cell lymphoma, Figure 2). Picolog inhibits cell growth and induces cell death in a wide range of lymphomas and leukemias.

### Picolog induces apoptosis through activation of PKC

**Figure 3:**
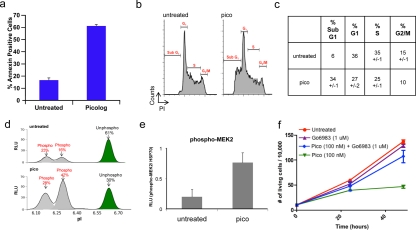
(a) Annexin-PE staining increased after treatment of 4188 cells with picolog (100 nM) for 48 hours (n=2). (b) Cell cycle analysis after 48 hours for untreated 4188 cells (n = 2) as well as cells treated with 100 nM picolog (n = 2). (c) Values for cell cycle analysis. (d) NIA traces demonstrating relative ratios of phosphorylated and unphosphorylated isoforms of MEK2 in untreated 4188 cells (top) and 4188 cells treated with picolog (100 nM, bottom). X-axis: Isoelectric point (pI). Y-axis: Relative Light Units (RLU). (e) NIA quantification of MEK2 phosphorylation, normalized to loading control (HSP70). Samples were analyzed in duplicate and graphed +/− SEM. (f) Cell growth over time for untreated 4188 cells as well as those treated with Go6983 (1 μM), picolog (100 nM) and picolog (100 nM) + Go6983 (1 μM). Each point represents the average of two values +/− SEM. P value (two-tailed unpaired t test): 0.042 (Pico (100 nM) + Go6983 (1 μM) vs. Pico (100 nM), 48 hour time points).

We next sought to characterize the mechanism through which picolog exerts its activity. Bryostatin is known to induce apoptosis by activation of PKC effectors, but other targets have also been proposed [29]. To determine if picolog treatment induced apoptosis, annexin staining was performed. Following treatment with picolog for 48 hours, significant apoptosis, but not cell cycle arrest, was induced compared with untreated control (p = 0.002) (Figures 3a-c). Previously it was demonstrated that bryostatin can increase MEK phosphorylation via activation of the PKC pathway. Using a novel nanofluidic proteomic immunoassay (NIA) [45-46] that enabled highly quantitative detection of relative phosphorylation and protein expression, we examined if picolog could also activate PKC and induce MEK phosphorylation. NIA revealed two distinct phospho-isoforms of MEK2 (Figure 3d). Prior to treatment, only 39% of MEK2 was in a phosphorylated state, with 61% of total MEK2 in the unphosphorylated state. Following treatment with picolog (100 nM) for 24 hours, relative MEK2 phosphorylation increased to 70%. Total MEK2 phosphorylation (normalized to loading control) also increased (Figure 3e, p = 0.04). To determine if growth inhibitory activity of picolog was due to PKC activation, the broad spectrum PKC inhibitor Go6983 was administered alone or together with picolog (Figure 3f). PKC inhibition alone did not decrease cell growth. However, co-administration of Go6983 with picolog abrogated the activity of picolog, indicating that picolog induces apoptosis through activation of the PKC pathway.

### Picolog inhibits tumor growth in vivo

**Figure 4:**
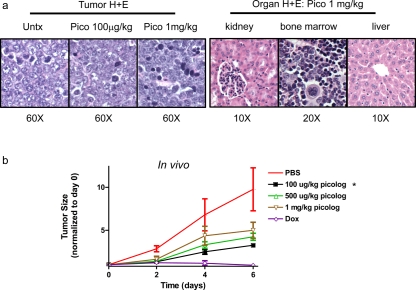
(a) Histology sections stained with hematoxylin and eosin for tumor tissues and organs collected at time of necropsy. (b) Tumor growth over time for untreated mice (n = 3), mice treated with picolog (100 μg/kg n = 4, 500 μg/kg n = 4, 1 mg/kg n = 3) or doxycycline n = 4. *indicates statistical significance relative to PBS group (two-tailed unpaired t test: p = 0.005 (100 μg/kg, day 2), 0.04 (100 μg/kg, day 4), and 0.03 (100 μg/kg, day 6)).

Picolog's therapeutic efficacy was investigated *in vivo*. Mice were injected subcutaneously with the 4188 transgenic lymphoma-derived cell line. Cohorts of mice were treated with picolog (100 μg/kg, 500 μg/kg, 1 mg/kg once daily, respectively) or doxycycline (to inhibit transgenic MYC via the Tet-system). None of the mice treated with picolog experienced any noticeable adverse side effects, including weight loss, immobility, or death. Upon histologic examination, no evidence of organ toxicity could be found in mice treated at the highest dose, 1 mg/kg (Figure 4a). Picolog was found to inhibit tumor growth in a reverse dose-dependent manner, with the 100 μg/kg dose eliciting the best response (Figure 4b). Statistically significant tumor growth inhibition (p < 0.05) was also observed for the 100 μg/kg treatment group. Overall, picolog was well tolerated and demonstrated promising therapeutic efficacy *in vivo*.

## DISCUSSION

Bryostatin 1 is a promising therapeutic lead that exhibits a unique portfolio of biological activities for the treatment of cancer and other diseases [1-4]. More recently, it has advanced to clinical trials for the treatment of cancer and of Alzheimer's disease. Its further advancement is hampered however by its poor availability from natural sources. Its severely limited abundance, along with its complexity, has also limited access to comparably effective structural analogs, especially agents that might be tuned to minimize off-target clinical toxicities. We have used synthesis-informed design to create simplified bryostatin analogs that could supply clinical needs, especially if they exhibit the exceptional potency and thus low dose requirements of bryostatin 1. This current study was designed to determine whether a lead analog in this series is effective against *in vivo* models of cancer. We found that picolog, a synthetically available analog of bryostatin, has superior activity relative to the natural product bryostatin 1. *In vitro*, picolog is remarkably potent for inhibition of growth of MYC-induced lymphoma and leukemia cells, with activity seen at concentrations as low as 50 nM *in vitro*. Picolog induced apoptosis *in vitro*, a result that is consistent with a previous report using picolog to treat Toledo non-Hodgkin's lymphoma cells [29]. Further, when dosing at 250 nM, the therapeutic efficacy of picolog was similar to targeted inhibition of the MYC oncogene. In mice treated with picolog *in vivo*, treatment with 100 μg/kg resulted in significant tumor growth inhibition. We have determined the optimal therapeutic dosing both *in vitro* and, for the first time, *in vivo* in a preclinical model of T-cell lymphoma.

Picolog's promising therapeutic efficacy *in vivo* raises several points for discussion. Importantly, picolog was found to be well tolerated by mice at all dosages tested. Therapeutic efficacy using as little as 100 μg/kg suggests that picolog is highly potent, especially in comparison with standard chemotherapeutic agents that are typically administered in mice in the 1-500 mg/kg range. Interestingly, picolog inhibited tumor growth in a reverse dose-dependent manner. The reverse dose dependence observed is not unexpected as overstimulation of PKC at high concentrations could result in degradation or downregulation of the enzyme over time, a process which is also known to occur in a dose-dependent manner with bryostatin 1 and further underscores the similar activities of the two agents [47]. Continued *in vivo* investigation of picolog as well as other bryostatin analogs is warranted given the efficacy and tolerability that we observed.

One mechanism of picolog's activity is thought to involve the PKC pathway. Picolog is known to be a high affinity ligand for PKC (*K*_i_ = 0.25 nM) [27]. To measure activation of MEK in the PKC pathway we developed the use of a novel nano-immunoassay (NIA) that enabled highly quantitative detection of relative phosphorylation and protein expression. NIA revealed two distinct phospho-isoforms of MEK2 in the PKC pathway that were induced upon picolog treatment. Having the ability to make NIA proteomic measurements using nanoscale amounts of specimen as the drug goes through additional preclinical and future clinical studies would enable the development of novel clinical biomarkers for monitoring therapeutic efficacy.

In our tumor model, we have determined that picolog induces PKC activation in rapidly dividing lymphoma cells. Other targets that activate this pathway, such as Ras, may also cooperate in the mechanism of tumor growth inhibition. Abrogation of activity by PKC inhibition is consistent with the pathways known to be directly modulated by bryostatin 1 and analogs [29]. Recent studies suggest that bryostatins also have the ability to activate the PKC pathway in normal lymphocytes [11, 13, 48-50]. Thus, in addition to direct anti-neoplastic effects on tumor cells, picolog could also have anti-neoplastic effects on host immune cells. Further studies of therapeutic and immunotherapeutic activity of picolog across a range of tumors including hematologic and solid malignancies should be performed to further elucidate its mechanism of action.

While this study focused on picolog, it is noteworthy that over 100 other analogs have been produced in our series and more than 30 exhibit PKC potencies comparable to or better than bryostatin 1. Significantly, while exhibiting single digit nanomolar or picomolar potencies, selected members of this analog library exhibit differential PKC isoform selectivities, thus offering the potential for selective targeting and, consequently, therapeutic optimization [51]. Along with exciting clinical results involving bryostatin 1 in combination with other agents for the treatment of lymphoma [11, 13], our findings help to substantiate that additional B- and T-cell hematopoietic malignancies could be candidates for the continued *in vivo* investigation of picolog and bryostatin analogs that can be supplied and tuned for activity.

We have shown that the simplified bryostatin analog, picolog, which can be synthesized in quantities needed for clinical advancement, has superior *in vitro* activity to bryostatin 1 in inhibiting growth and inducing apoptosis in MYC-induced lymphoma. For the first time, we also have shown that this analog is effective *in vivo* in an animal model of cancer. When extrapolated to a clinical setting, dosages of picolog sufficient to treat patients could be readily supplied through synthesis. Therapeutic activity of picolog could be explored across a range of tumors including hematologic malignancies tested in this work, as well as a range of solid tumors known to have PKC dysregulation. Future efforts will focus on the continued preclinical investigation of bryostatin analogs, including further refinement of single agent *in vivo* activity as well as the examination of clinically relevant combination regimens and immunotherapeutic activity.

## MATERIALS AND METHODS

### *In Vitro* Cell Growth and Cell Cycle Assays

Cell line growth conditions. Cell lines 4188 and 6780 were derived by the Felsher Laboratory from transgenic mice conditionally over-expressing human c-MYC using the Tet-system [34-37, 39-41] and maintained between 100,000 cells/mL and 2,000,000 cells/mL in RPMI 1640 media (Gibco, 10% fetal bovine serum, 1% penicillin/streptomycin added, 0.1% beta-mercaptoethanol added) in a 37 °C incubator (5% CO_2_). Jurkat cells (ATCC) and CA-46 cells (ATCC) were maintained between 100,000 cells/mL and 1,000,000 cells/mL in RPMI 1640 media (Gibco, 10% fetal bovine serum, 1% penicillin/streptomycin added). Cell lines are routinely authenticated by flow cytometry phenotyping and gene expression studies.

*In vitro* administration of picolog. In a 6-well plate (Costar, low evaporation lid), 4 mL cells in log-growth phase (100,000-150,000 cells/mL) were added to each well. Next, agents (bryostatin 1, picolog, Go6983 inhibitor) were added either as stock solutions in DMSO or as solutions in room temperature media (RPMI 1640) diluted from the DMSO stock solutions. Go6983 was purchased from Sigma Aldrich and diluted into DMSO stock solutions upon receipt. Each experimental group was dosed in duplicate, and untreated wells received DMSO in an equal concentration to the most concentrated treatment group (final DMSO concentration < 0.1%). Doxycycline was added as a solution in 0.9% phosphate buffered saline. In the case of the PKC inhibitor (Go6983) combination dosing with picolog, picolog was dosed first followed immediately by the inhibitor. Cells were counted at 24-hour intervals using a hemocytometer and trypan blue staining. Values were obtained in duplicate for each control and treatment group.

The data for these experiments was plotted using GraphPad Prism version 5.0 in an XY graph. Multiple hemocytometer counts for the same well were averaged. The data was plotted as the (number of living or dead cells)/10,000 over time. Points represented the average of the two duplicate values from each treatment group +/− the standard error of the mean.

Cell cycle analysis. Cells were washed once with PBS, then resuspended in 70% EtOH/PBS for fixation and permeabilization, and stored at −20 °C. At the time of flow cytometry analysis, cells were washed once with PBS and resuspended in 20 μg/mL propidium iodide in PBS (Invitrogen). 10,000 events per sample were collected on a FacScan Flow Cytometer (BD Biosciences) and analyzed using CellQuest (BD Biosciences).

Apoptosis analysis. Cells were washed once with PBS, resuspended in 200 μL of 1X binding buffer (BD Pharmingen), and stained with 20 μL Annexin V-PE (BD Pharmingen)/20 μL 7-AAD (BD Pharmingen). 10,000 events per sample were collected on a FacScan Flow Cytometer (BD Biosciences) and analyzed using CellQuest (BD Biosciences).

### Nanofluidic Immunoassay (NIA) for Phosphoprotein Quantification

Samples were run in duplicate. Lysates for the isoelectric focusing experiments were generated by taking an aliquot of 100,000 cells from treatment wells in a 6-well plate (for the cell growth/death versus time experiments). In the case of the solid tumor tissue, lysates were generated from the filtrate of the tumor homogenization. Cells were lysed in 10 μL of MPER lysis buffer (Invitrogen) containing protease and phosphatase inhibitors (Invitrogen). After 30 minutes on ice, protein lysate was collected by centrifugation at 14,000 RPM at 4° C for five minutes. For each sample well in the 96-well plate, 12 μL pre-mix (1% standard (4.9, 7.0, 7.3) isoelectric point 2-11, ProteinSimple), 6 μL supernatant, and 6 μL HNTG were added. 50 μL/well of each primary antibody solution (1:100 MEK2 antibody (Cell Signaling), 1:100 HSP70 antibody (Santa Cruz)) was diluted in antibody dilution buffer (ProteinSimple). 100 μL/well was added for each secondary antibody in another row on the 96-well plate (1:500 dilution for anti rabbit-HRP (Santa Cruz), 1:250 dilution for anti mouse-HRP (Santa Cruz)). A 96-well plate loaded with sample, primary, and secondary antibodies was placed into the Firefly 3000 instrument (previous platform generation to the current Nanopro1000, ProteinSimple). Compass software was used to set up and implement the run according to manufacturer protocols, to identify the peaks, quantitate the areas under the peaks, calculate the percent phosphorylation, and generate trace images. NIA best-fit traces are graphed showing isoelectric point (pI, x-axis) vs. relative light units (RLU, y-axis). Quantification of MEK2 phosphorylation is obtained by summing the area under the curve of phosphorylated peaks for each sample, then dividing the sum by the area under the curve for loading control (HSP70) in order to normalize for loading. Normalized MEK2 phosphorylation is graphed (RLU phospho-MEK2/ HSP70, y-axis) +/− SEM.

### *In Vivo* Study Using Picolog

Ten female FVBN mice were divided into treatment cohorts: untreated, doxycycline (100 μg/ml) control, picolog 100 μg/kg, picolog 500 μg/kg, and picolog 1 mg/kg. Mice were housed and maintained in the Stanford University Research Animal Facility per approved APLAC protocols. On day zero, each mouse received bilateral subcutaneous flank injections of 800,000 4188 cells per flank. Tumor size was monitored via caliper measurement and, on day six, treatment began. Mice from each group received 100 μL i.p. injections once daily. The untreated control mice received injections of 0.9% PBS with <0.1% DMSO. The doxycycline group received treatment in their drinking water. For the picolog treatment groups, a solid sample was dissolved in DMSO to make a 4 mM stock solution. This stock solution was diluted into 0.9% PBS, and mice received once daily 100 μL i.p. injections. Over the course of the study, tumor size was monitored via electronic caliper measurements. Mice were also weighed every 48 hours. Once tumor sizes reached ~ 20 mm^2^, mice were sacrificed by asphyxiation with CO_2_ gas. Immediately after sacrificing the mice, the brain, kidneys, liver, spleen, heart, and lungs were harvested along with the tumor tissue. The organs were fixed in 95% ethanol for future analysis. Solid tumor tissue was flash frozen in liquid nitrogen and stored at −80 °C. The solid frozen tumor tissue was used to generate lysates for the isoelectric focusing experiments. The tumor growth curves (tumor size represents tumor volume (calculation = length × width^2^ × 0.52)) were generated using GraphPad Prism version 5.0.
